# Towards Low Cost and Low Temperature Capacitive CO_2_ Sensors Based on Amine Functionalized Silica Nanoparticles

**DOI:** 10.3390/nano9081097

**Published:** 2019-07-31

**Authors:** Jamila Boudaden, Armin Klumpp, Hanns-Erik Endres, Ignaz Eisele

**Affiliations:** 1 Fraunhofer Research Institution for Microsystems and Solid State Technologies EMFT, Silicon Technologies and Devices, Hansastrasse 27d, 80686 Munich, Germany; 2 Institute of Electronic and Sensor Materials, TU Bergakademie Freiberg, Gustav-Zeuner-Str. 3, 09599 Freiberg, Germany

**Keywords:** CO_2_ chemical sensor, hybrid organic–inorganic materials, nanoparticles, functionalization

## Abstract

Hybrid materials based on inorganic particles and an organic polymer were developed and used as an efficient sensing material for carbon dioxide (CO_2_). The sensing material consists of fumed silica that is functionalized with an organic polymer, polyethylenimine, by means of the impregnation method. The organic polymer is effectively immobilized around the silica nanoparticles and confirmed by infrared spectroscopy. Thermogravimetric analysis proves the thermal stability of the sensing material. CO_2_ capacitive sensors operating at temperatures lower than 70 °C were fabricated by depositing a thin layer of hybrid sensing material on interdigitated gold electrodes. Impedance spectroscopy explored the sensing capability of the hybrid organic–inorganic material towards CO_2_ in the presence of different relative humidity levels, as well as its stability and reversibility. This strategy to couple organic and inorganic materials as a sensing layer for CO_2_ paves the way for the design of a low-cost CO_2_ sensor.

## 1. Introduction

Our atmosphere contains a concentration of 400 ppm CO_2_. A high level of CO_2_ in a closed building may lead to serious health problems for occupants, especially if the concentration exceeds 5000 ppm. CO_2_ concentrations between 1000 and 2000 ppm during 30 to 90 min in meeting rooms can be acceptable [1]. The organization OSHA (Occupational Safety and Health Administration) has set an exposure limit to CO_2_ of 5000 ppm and 30,000 ppm over an eight-hour and a 15-min period, respectively. The European standard has even set a shorter exposure time limited to 15 min in the presence of 15,000 ppm CO_2_. So far, several companies have communicated great interest in the development of a cost-effective chemical CO_2_ sensor that enables monitoring carbon dioxide amount in the surrounding atmosphere. Additionally, monitoring the “Indoor Air Quality (IAQ)” in modern buildings with ventilations requires CO_2_ sensors to ensure the wellbeing of the occupants by adapting the ventilation rates to their needs. CO_2_ sensors will optimize ventilation rates to lower the energy dissipation [2] because a continuous ventilation of thermally isolated buildings contributes to the increase of the overall energy consumption [3]. In addition to indoor applications, CO_2_ sensors are also desired for early detection of human disease, capnography and emissions by industrial combustion processes [4,5]. Although several applications need the detection of CO_2_, the chemical inertness of the carbon dioxide molecule is a bottleneck in developing cost-effective chemical sensors.

Optical detection method based on the non-dispersive infrared (NDIR) method is one well-known way and established concept in detecting gases [6].Carbon dioxide can be easily identified by infrared spectroscopy because it is an infrared active molecule, which absorbs a 4.24 µm wavelength. Commercial CO_2_ sensors based on the NDIR principle are accurate, allow fast measurements and have a good long-term stability. The downsides of the optical sensors are the unscalable size, power consumption and high commercial costs. Conventional CO_2_ sensors based on solid-state electrolytes, metal oxides, were also studied [7,8,9,10,11,12,13,14]. The materials used for CO_2_ sensors based on solid-state electrolytes and metal oxides inevitably show a huge cross-sensitivity to other gases and require operating temperatures higher than 100 °C to reach an acceptable sensitivity. Recently, tandem p-i-n diodes on a planar p-type (100) silicon and nanorods silicon substrates with Ag additive were realized to detect CO_2_ [15]. Once CO_2_ is in contact with the diode device, Pd/SnO_2_/Al_2_O_3_/CuO/Ag/SnO_2_/Al_2_O_3_/CuO/p-Si/Al, the molecules are adsorbed on the surface of the SnO_2_ sensing layer and catalyzed to carbonate (CO_3_^2−^) by the palladium electrode at 150 °C. The repeatability of the sensor was tested in four cycles under various CO_2_ concentrations and showed a similar transient behavior. The response and the recovery times of the device are 42 s. 

At present, an alternative concept and sensitive materials to detect CO_2_ are still desired. The focus on using polymer films as gas sensing layers (e.g., for CO, CO_2_ and NO_2_) is one promising and attractive choice due to their low cost process fabrication and low reaction temperature [16]. During absorption and desorption processes, gas molecules react with functional groups imbedded in polymers and a change in the electrical bulk property (such as conductivity, capacitance or dielectric permittivity) can be depicted [17,18,19,20]. Amino groups are the most-used and studied functional groups to detect the presence of CO_2_. An interaction of CO_2_ and amino groups is explained by the “Hard Soft Acid Base (HSAB) theory” [21,22]. CO_2_ is considered as a hard acid, while primary and secondary amines are hard bases. Primary and secondary amines react reversibly with CO_2_ by ionic interaction to form carbamate products, while tertiary amines effectively react with CO_2_ only in the presence of water to form bicarbonate [23,24]. Several types of amine-based polymers have been synthesized and their potential feasibility in sensing CO_2_ was demonstrated. Heteropolysiloxanes prepared by a copolymerization of monomeric alkylsilane and aminoalkylsilane, such 3-aminopropyltrimethoxysilane (AMO) and propyltrimethoxysilane (PTMS) [25,26,27,28,29,30,31], and poly(y-aminopropylethoxy mixed with propylethoxysiloxane (PAPP) or octadecylethoxysiloxane (PAPO) [32,33] were intensively studied. To further reduce the cross-sensitivity of an amine-based polymer material to humidity, a hydrophobic polymer matrix is often added [24,34].

Polyaziridine is another type of amine-based polymer with a repeating unit of an amino group and two carbon aliphatic spacer -[NH-CH_2_CH_2_]-. Polyaziridine, called also polyethyleneimine (PEI), can be found as a branched or a linear structure, and consists of primary, secondary and tertiary amine groups. Polyethyleneimine is used to functionalize an array of solid sorbents particularly employed in the storage of CO_2_ due to their a high CO_2_-capture capacity [35,36]. In the field of CO_2_ sensors, PEI was explored using different transduction mechanisms. A PEI-coated surface acoustic wave transducer (SAW) showed a sensing ability to the presence of H_2_O and CO_2_, accompanied with a baseline drift of the recorded signal and an unpreventable degradation of their sensing ability to CO_2_ [37]. A quartz microbalance transducer coated with a PEI layer showed sensitivity to CO_2_ at an operating temperature of 60 °C, with an unavoidable cross-sensitivity to water [30]. It was found that the PEI/Starch nanocomposite thin film, which can be described as a mixture of two organic compounds, exhibited a better CO_2_-sensing property than the thin layer of pure PEI [38].

We have noticed a limited effort dedicated to the exploitation of an alternative sensitive material to CO_2_ [17,18,20]. CO_2_ sensing materials studied are based on organic materials and have a lack of stability, selectivity, sensitivity, reversibility and in some cases necessitate a high temperature of operation. In this paper, we report the synthesis of the hybrid PEI/Silica nanoparticles and deposition of a thin sensing layer on interdigitated gold electrodes. The as-fabricated transducer and hybrid sensing material exhibit a good sensitivity towards CO_2_. A real-time continuous measurements of the prepared CO_2_ capacitive sensor enables exploring kinetic data in the presence of both CO_2_ and relative humidity. The results of experiments indicate that the hybrid organic–inorganic CO_2_ sensing layer shows the best sensor performance obtained to date for in door application, such as reproducibility, stability, sensitivity and linearity. 

## 2. Experimental

### 2.1. Fabrication of Transducers and Calibration of the Resistive Heater

A pair of interdigitated microelectrodes was produced by standard photolithography on a 620 µm thick glass substrate. After cleaning the glass substrate wafer, a thin adhesion layer of TiW (40 nm) followed by a thick gold layer (140 nm) were deposited by the sputtering technique. The electrodes, a resistive heater and a temperature sensor, were patterned by the photolithography technique using the desired mask. The heater and the temperature sensor are made around the gold fingers of the interdigitated transducer (IDT) [39]. Each chip featured two interdigitated gold electrodes, each consisting of 83 fingers and filling an area of 2 × 2 mm^2^. Finger widths and pitches were set to 6 and 12 µm, respectively. The wafer was diced into 4.7 × 3.9 mm^2^ large individual chips, then mounted on an adapted printed circuit board (PCB) and finally gold wire bonded thereto. 

Until today there is an absence of complete information about the adsorption kinetics of CO_2_ molecules on amine functionalized silica nanoparticles. For this purpose, we developed a standard method, which controls the temperature of operation of the CO_2_ sensor. The resistance of the temperature sensor on bare IDT, before coating with a sensing material, was systematically measured by introducing them into an oven, heated from 25 to 100 °C. The same sensors were heated by applying a voltage to the heater, from 1–6 V, and depicting the temperature sensor resistance. 

This calibration procedure allowed us to establish the relationship between the voltage applied to the heater and the resistance of the temperature sensor to determine precisely the desired operation temperature of the CO_2_ sensor. By applying a defined voltage to the heater, the temperature deviation (within the area occupied by the interdigitated electrode) from the desired operation temperature was around 1 °C. This value was determined by an infrared camera [34]. The small discrepancy between the measured capacitance of the uncoated transducer, called base capacitance, the heater resistance and the temperature sensor of different chips from the same wafer was due to the inhomogeneity of the sputtered gold layer thickness on a 150 mm glass substrate and the difficulty in controlling accurately the wet etching process of the sputtered gold layer on the glass substrate.

### 2.2. Synthesis of the Hybrid Organic–Inorganic Sensing Material

Generally, amine functionalized silica hybrid materials are synthesized by grafting (a chemical bond is formed between the amine and the support) and impregnation (a physical mixture of the amine and the support). Hybrid amine–silica composite prepared by impregnation for making CO_2_ sorbents yielded a higher amine content and higher CO_2_-adsorption capacity than grafting [40]. In the present work, we adopted the impregnation method to synthetize the polyethyleneimine/silica hybrid sensing material. First, the desired amount of PEI was dissolved in 50 mL ethanol under stirring with a magnetic stirrer. After a complete dissolution of PEI, 5 g of silica nanoparticles were then added. A continuous stirring of the mixture took place at room temperature for 30 min. Subsequently, the product was dried for 2 h at 50 °C under vacuum (<10 mbar) so as to obtain a white powder. The obtained materials were named PEI@silica (*a*), where *a* refers to the size in nm of inorganic silica nanoparticles and the PEI loading is fixed to 50 wt. % of the total composition.

### 2.3. Coating of the Hybrid Sensing Material onto the Interdigitated Transducer

**Figure 1:**
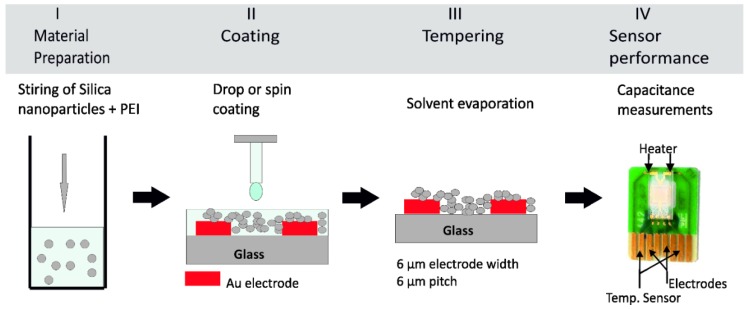
Scheme of the preparation of a capacitive CO_2_ sensor in four steps. (Step **I**) Magnetic stirring of the organic PEI and inorganic Silica nanoparticles. (Step **II**) Spin coating or drop coating of the PEI@silica resulting suspension onto a clean interdigitated transducer on glass substrate. (Step **III**) Tempering process at 120 °C for 15 min to ensure a complete solvent evaporate from the sensing layer. (Step **VI**) Capacitive measurement of the prepared CO_2_ sensor.

Figure 1 describes the steps necessary for preparing a sensing layer for CO_2_ on the interdigitated transducer sensor. Firstly, the powder of hybrid sensing material is dissolved in ethanol solvent to obtain a homogenous mixture, see Figure 1, step I. The wetting of the transducer surface becomes difficult once the mixture viscosity is higher. As it is desired to coat the planar interdigitated electrodes, the wettability between the mixture and the substrate is an important parameter to be considered in order to get a homogeneous thin film. Decreasing the mixture viscosity results in a good wetting behavior, permitting the formation of a homogeneous film on the gold electrodes. A layer of sensing material of 4 µm was spin coated on the top of planar electrodes, see Figure 1, step II. The coated transducer underwent a drying step at 120 °C for 15 min, see Figure 1, step III. Then, the sensors were ready for electrical characterization as in Figure 1, step IV.

### 2.4. Gas Mixing Apparatus and Capacitance Measurements of Coated Interdigitated Transducers

A reliable gas mixing apparatus, which is a part of a gas measurement setup, is an important system for characterizing gas sensors under desired gas mixtures. The dynamic volumetric method, described in Reference [41], was adopted to generate a defined gas mixture in a reproducible way. For this study, the gas mixing apparatus was able to generate a constant level of absolute humidity and CO_2_ concentration by selecting the adopted ratio between the volumes of humid gas or CO_2_ gas to the carrier gas, which is N_2_ or synthetic air. 

The electrical properties of the CO_2_ sensors were monitored by impedance spectroscopy using a Solartron 1260 gain-phase frequency analyzer (model 1260A) controlled by a PC, permitting automated data collection. Up to 10 chips were characterized in the same test chamber under the same gas environment. The impedance measurements can be carried out at different temperatures (from RT to 200 °C) by heating up the chip using the integrated heater around the electrodes. For the present study, the impedance parameter was recorded at 40 kHz. Up to 10 chips could be placed into a cylindrical stainless steel chamber (sensor chamber), which is connected to the gas mixing apparatus [39]. Consequently, they were simultaneously characterized under the same gas environment conditions. A radial distribution of the gas and isolating wall between the 10 chips ensured a continuous and controlled flow of the gas. The sensor is subjected to a dynamic change of relative humidity levels and CO_2_ concentrations and its capacitance is monitored. 

### 2.5. Analytical Methods of As-Prepared Raw Hybrid Materials

Fourier transformed infrared (FTIR) spectra of as-prepared hybrid materials were recorded on a PERKIN ELMER Spektrum BX II FTIR spectrometer at room temperature, using an attenuated total reflectance unit.

The N_2_ adsorption/desorption isotherms of as-prepared hybrid materials were recorded with a Coulter SA 2100. Before adsorption/desorption experiments, the samples were degassed at 150 °C for 120 min.

Thermo-analytical measurements (TGA) were performed with a Setaram Thermoanalyzer TG-DTA92 on the as-prepared hybrid materials. The measurements were conducted in argon inert atmosphere employing alumina crucibles. The sample was heated from room temperature to 400 °C with a rate of 10 °C min^−1^.

## 3. Results and Discussion

### 3.1. Nitrogen Sorption Characteristic of As-Prepared Raw Hybrid Organic–Inorganic Materials

**Figure 2:**
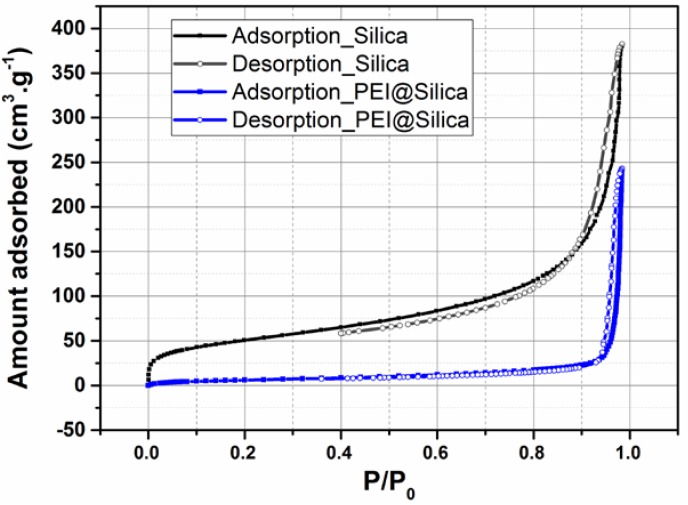
Nitrogen adsorption (closed symbols) and desorption (open symbols) isotherms for PEI@silica (*100 nm*) sensing material.

The N_2_ adsorption/desorption isotherms of silica before and after loading them with PEI were measured to evaluate their surface area and pore volume. As shown in Figure 2, the bare silica exhibited high N_2_ uptakes. The corresponding BET surface area and pore volume (V_p_) were calculated to be 189.74 m^2^ g^−1^ and 0.741cm^3^ g^−1^, respectively. After loading of PEI into silica, the N_2_ uptake, surface area and pore volume decreased dramatically. As shown in Figure 2 and Table 1, the surface area and pore volume of PEI@silica were 24 m^2^ g^−1^ and 0.0058 cm^3^ g^−1^, respectively. Actually, the reduced pore volume indicated that the PEI was loaded into the silica-free area or between the formed agglomeration of several silica nanoparticles [42].

### 3.2. Thermal Stability Analysis of the As-Prepared Raw Hybrid Organic–Inorganic Materials

**Figure 3:**
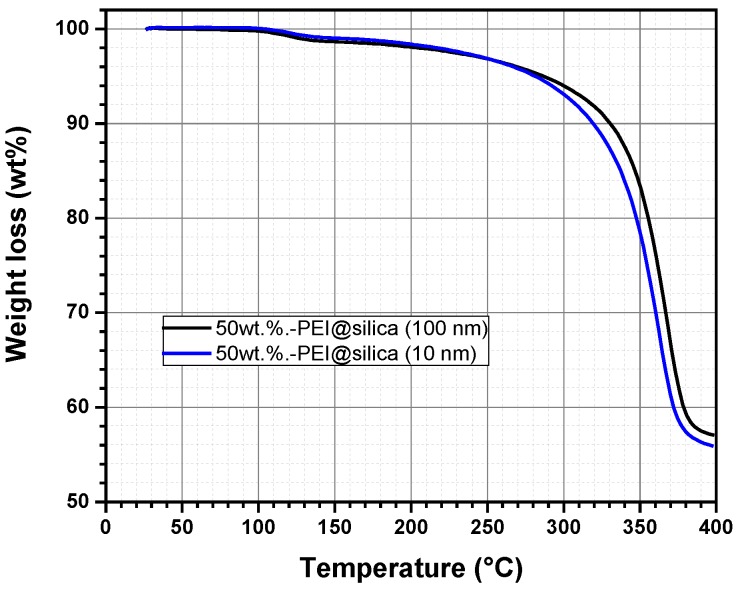
Thermogravimetric analysis (TGA) of the sensing material PEI@silica (*a*) under Argon flux.

It is known that polymeric materials are easily decomposed once heated up to a temperature exceeding 100 °C. The thermal degradation causes a change in the physical, mechanical, or electrical properties due to the polymer mass loss [43]. For this reason, thermogravimetric analysis (TGA) has been used to study the thermal stability of the prepared hybrid sensing materials PEI@silica, using two silica nanoparticle sizes (10 nm and 100 nm). Figure 3 represents the TGA curves of the prepared hybrid materials, PEI@silica (*10 nm*) and PEI@silica (*100 nm*), which were dried under vacuum before loading into a ceramic crucible for TGA measurements. Then the temperature is increased from 20 to 400 °C with a heating ramping rate of 10 °C min^−1^ under Argon flux. 

The hybrid materials, PEI@silica (*10 nm*) and PEI@silica (*100 nm*), show the same decomposition behavior, which is dependent on the temperature but independent on the size of the used silica. Generally, it is known that silica materials undergo no decomposition under a thermal process (T < 800 °C) [36]. But, the decomposition of the PEI polymer compound is almost completed when the temperature reaches 400 °C. From room temperature up to 130 °C, an initial weight loss of 2% is probably attributed to the release of physically and chemically adsorbed water and solvent on the PEI@silica sensing material. At temperatures above 130 °C till 250 °C, the volatilization and decomposition of PEI molecules located on the outside surface of the silica can also explain the depicted light thermal degradation [44]. Besides, the de-hydroxylation of silica might contribute to the weight loss of PEI–silica materials [45]. At temperatures above 250 °C till 375 °C, the weight loss of PEI–silica materials is mainly due to the volatilization and decomposition of PEI molecules located in the shallower parts of the pores. At 375 °C, a complete decomposition of the PEI polymer is reached. This means that the resulting product after decomposition should be silica with a final mass weight of 50%, which is in accordance with the measured value of 55% by TGA. The same observation was reported for silica adsorbents impregnated with different PEI contents [36]. Based on the TGA results, PEI@silica (*10 nm*) and PEI@silica (*100 nm*) materials exhibit relatively good thermal stability below 250 °C due minimal volatilization of PEI molecules. Hence, a thin layer of organic materials is formed around the silica nanoparticles and/or soaked deeply between the inorganic silica nanoparticles, because the silica nanoparticles tend to form clusters. The prepared hybrid material has good thermal stability in comparison to pure organic polymeric layers [46].

### 3.3. FTIR Analysis of the As-Prepared Raw Hybrid Organic–Inorganic Materials

**Figure 4:**
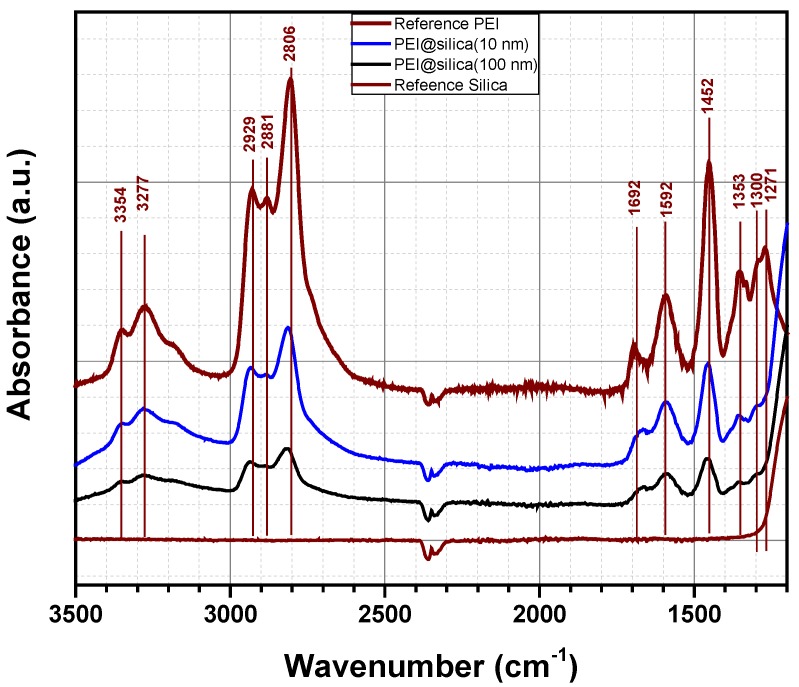
Absorbance spectra of pure PEI, pure silica and PEI@silica hybrid organic–inorganic materials.

Figure 4 shows the FTIR spectra of PEI@silica materials in the range of 1200–3500 cm^−1^ to confirm the functionalization of the silica by the amino groups. The spectra of pure PEI and pure silica materials are also included for comparison. The IR spectrum of pure PEI shows absorption bands at 3354, 3277 and 1592 cm^−1^, which can be assigned to asymmetric NH_2_ stretch (υ_as_ NH_2_), symmetric NH_2_ stretch (υ_s_ NH_2_) and NH_2_ deformation (δ NH_2_) of the hydrogen bonded amino group, respectively. The large absorption band at around 3300 cm^−1^ is due to an overlap of NH stretch of secondary amine (υ NH) and symmetric NH_2_ stretch (υ_s_ NH_2_). Besides, absorption bands related to CH_2_ stretch (2881 and 2929 cm^−1^) and CH_2_ deformation (1452 and 1353 cm^−1^) were also observed. The same absorption bands of 3354, 3277 and 1592 cm^−1^, assigned to NH_2_ amino groups, were detected for prepared PEI@silica materials by other groups. Qi et al. prepared nanocomposite sorbents based on polyethyleneimine supported on mesoporous silica hollow capsules (PEI@SBA), which were characterized by FTIR spectroscopy [47]. They found out that after exposing PEI@SBA to CO_2_, new absorption bands appeared at 1650, 1540 and 1407 cm^−1^, which can be assigned to N–H deformation in ${\mathrm{RNH}}_{3}^{+}$ stretch and NCOO skeletal vibrations due to the formation of carbamate. Two other broad bands at 2460 and 2170 cm^−1^ are due to a chemically absorbed CO_2_ species to the PEI@SBA material. Kim et al. investigated the formation of urea in PEI/SiO_2_ materials by FTIR after temperature sweep cycles for 20 times at 40 °C as the absorption temperature, and 130 °C as the desorption temperature. The IR spectra indicated the formation of both cyclic and open chain urea products [48,49]. The cyclic urea is formed via a chemical reaction between one molecule of CO_2_ and two amines within a single molecule (intra-molecular reaction). The corresponding adsorption bands were located at 1495 and 1708 cm^−1^ due to dehydrative condensation between one molecule CO_2_ and two amines from different molecules (inter-molecular reaction). The corresponding absorption bands were situated at 1560 and 1650 cm^−1^. In our study, the material PEI@silica was exposed to CO_2_ in the surrounding air in the laboratory for some weeks before performing FTIR analysis. As was confirmed by IR measurements, no additional absorption bands can be related to open chain urea or a formation of new products by a chemical absorption of CO_2_ on amine sites. It might be that the peak situated at 1700 cm^−1^ is related to the formation of cyclic urea, which results from the reaction of secondary amine groups with PEI. However, as no peaks related to the open chain urea, we believe that primary amine groups of PEI@silica are still active [49].

### 3.4. Sensing Performance towards CO_2_ of Coated Interdigitated Transducers

#### 3.4.1. Dynamic Sensing

**Figure 5:**
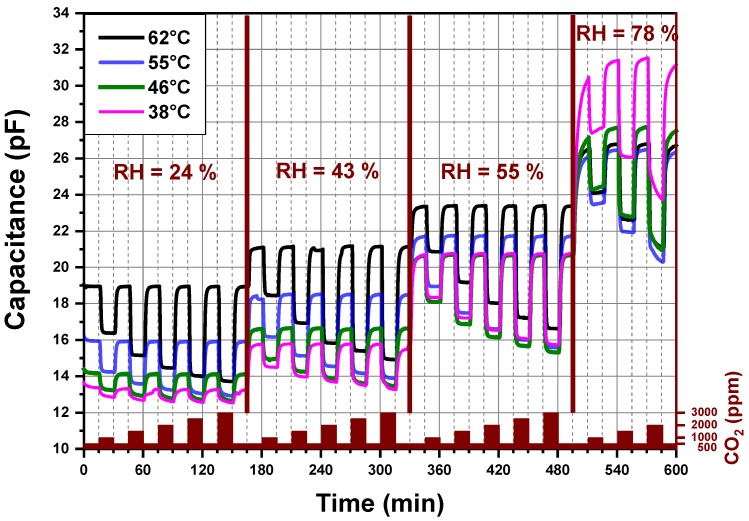
Capacitance versus time for a PEI@silica (*100 nm*)-based CO_2_ sensor at different operating temperatures and different relative humidity levels.

Figure 5 reports the dynamic-sensing response of the CO_2_ sensor measured by an impedance analyzer. The explored sensing layer is made of PEI@silica (*100 nm*). The imaginary part (capacitance) of the sensor impedance is plotted against the sensing time for different CO_2_ concentrations and relative humidity levels under atmospheric pressure. Additionally, the recorded four signals correspond to four different operating temperatures (38 °C, 46 °C, 55 °C and 62 °C) of the same CO_2_ sensor. The operating temperature is the temperature at which the CO_2_ sensing layer was heated locally. The sensor signal is measured first at 62 °C, then at 55 °C, then at 46 °C, then at 38 °C and finally again at 62 °C. The last measurement at 62 °C was done in order to confirm that no degradation was induced by the previous measurement and heating cycles of the sensor. The sensor capacitance shows changes, which were due to the exposition of the sensor to different CO_2_ concentrations and relative humidity levels mixed with synthetic air. If the operating temperature of the CO_2_ sensor is higher than 70 °C, the capacitance changes become less pronounced.

In Figure 5, four regions can be distinguished related to different relative humidity levels produced by the gas mixing apparatus of our setup. For each level of relative humidity, the sensor is alternatively exposed to different amounts of CO_2_ mixed with synthetic air, which are varied from 500 to 3000 ppm by steps of 500 ppm. The alternated exposure and recovery times of the sensor to different concentrations of CO_2_ were fixed to 15 min. The capacitance measured in the presence of 500 ppm CO_2_, at a given relative humidity, is considered as a background sensor capacitance. We observed no drift in the capacitance signal for a given and stable relative humidity less than 60%. At high (78%) relative humidity, the background capacitances of the CO_2_ sensor increase by decreasing the operating temperature from 55 to 38 °C. Besides, the 15 min exposure time of the CO_2_ sensor to 78% relative humidity at a stable CO_2_ concentration (500 ppm) is not enough to reach a stable background capacitance of the sensor independent of the operating temperature. An exposure time of at least 1h seems necessary. 

For a fixed relative humidity, the capacitance decreases by increasing the CO_2_ amount in synthetic air to reach a steady-state value. By decreasing the CO_2_ concentration to 500 ppm (background value) a fast increase of the capacitance is observed and the already measured background capacitance is again reached. Increasing the sensor operating temperature induces a systematic increase of the sensor background capacitance, except for a relative humidity higher than 60%. A fast and reversible behavior of the capacitance of the CO_2_ sensor without any hysteresis, under relative humidity less than 60%, is observed for an operating temperature above 46 °C and below 65 °C.

**Figure 6:**
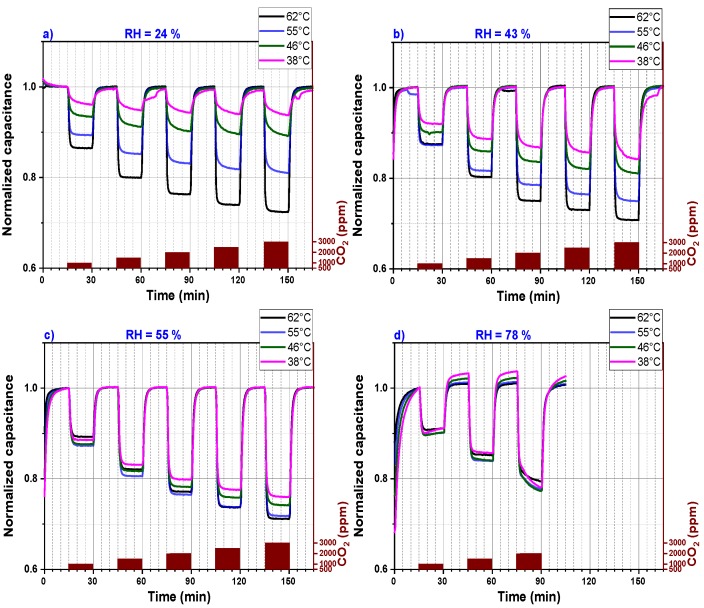
Capacitance (normalized to 1) versus sensing time of a PEI@silica-based CO_2_ sensor at different operating temperatures and for different relative humidities: (**a**) 24% RH, (**b**) 43% RH, (**c**) 55% RH and (**d**) 78% RH.

Figure 6 represents the data of Figure 5 in a simplified illustration. The sensor signals, which are plotted in Figure 5, are divided into four graphs (a, b, c and d) that represent CO_2_ sensors measured under four different relative humidity levels (24%, 43%, 55% and 78%), respectively. Each graph shows the capacitance measurement versus the sensing time for four different operating temperatures. Moreover, for each graph, the measured signal was normalized to one because at different operating temperatures, the CO_2_ sensor shows different background capacitances. This method permits an ease comparison of sensors performance operating at a defined relative humidity and different temperatures. The response time at temperatures above 46 °C is short as the sensor signal saturation is reached within less than 2 min. However, for an operating temperature below 46 °C, the CO_2_ sensor capacitance does not reach a stable value within 15 min, which explains the continuous small background drift of the CO_2_ sensor capacitance for relative humidity less than 60%. At a relative humidity higher than 55%, the sensor does not seem to reach a background capacitance within the 15 min exposure time. Longer exposure time is definitively necessary for the CO_2_ sensor operating at higher relative humidity. The sensor performance increases by increasing the sensor operating temperature if the relative humidity is below 60%. 

**Figure 7:**
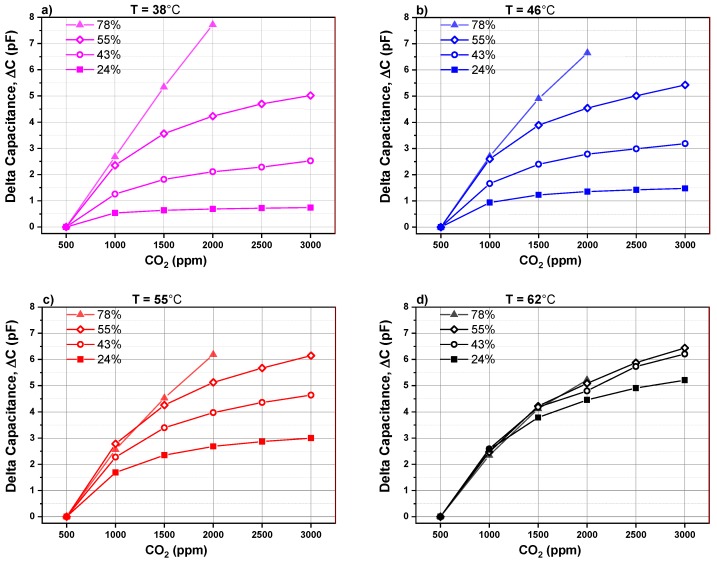
Capacitance change versus CO_2_ concentration for different humidity levels of a PEI@silica-based CO_2_ sensor at different operating temperatures: (**a**) 38 °C, (**b**) 46 °C, (**c**) 55 °C and (**d**) 62 °C.

The capacitance change is plotted in Figure 7 as a function of different CO_2_ concentrations mixed in synthetic gas and at different relative humidity levels. The absolute capacitance change (see Equation (1)) is defined as the difference between the background capacitance measured at 500 ppm CO_2_ and the capacitance monitored by introducing a higher concentration of CO_2_. This parameter will be called capacitance change for simplicity.

(1) $$Delta\;Capacitance,\Delta C=Capacitance\left(at\;500_{CO_{2}}ppm\right)-Capacitance\left(at\;X_{CO_{2}}ppm\right)$$

where *X_CO_*_2_ represents the CO_2_ concentration when it is higher than 500 ppm.

In the graphs, Figure 7a–d, the absolute capacitance change is represented as a function of CO_2_ concentration and relative humidity for four different operating temperatures (38 °C, 46 °C, 55 °C and 62 °C), respectively. The capacitance (at *X* ppm) corresponds to the value once the signal attains its minimum value and becomes stable. It has a negative sign due to the fact that an increase in the CO_2_ concentration in synthetic air decreases capacitance in regard to the baseline. 

At an operating temperature below 55 °C, the absolute capacitance change of CO_2_ sensor rises by increasing the relative humidity from 24% to 78%. We can also observe that the capacitance change behavior versus CO_2_ concentration can be divided into two regions. In region I, the capacitance change shows a quasi linear behavior by increasing CO_2_ concentration from 500 to 1500 ppm. The linear behavior is extended over a larger range till 2000 ppm if the humidity level is higher than 55%. In region II, from 1500 to 3000 ppm CO_2_, the capacitance change is less pronounced and tends to saturate. The presence of two regions is related to the fact that the CO_2_ sensitive layer has a limited number of available sites to adsorb CO_2_ adsorbent. If a 1500 ppm CO_2_ in the surrounding atmosphere is exceeded, the number of available sites for adsorbent decreases drastically, leading to saturation in the capacitance change. For a CO_2_ sensor operating at 62 °C, sensor signal is quite independent of the relative humidity. For operating temperature less than 62 °C, the CO_2_ sensor capacitance change is highly dependent on the relative humidity. The sensor signal increases by increasing the amount of water vapor molecules present while sensing CO_2_.

Theoretically, amine groups react with CO_2_ to form carbamate in the absence of water, as shown in reaction Equations (2)–(4). We have to notice that the adsorption capacity is limited due to the fact that one mole of CO_2_ reacts with two moles of amine groups. The reaction pathway between CO_2_ and the three types of amine groups (primary, secondary and tertiary) under dry conditions is given below:

(2) $${\mathrm{CO}}_{2}+2{\mathrm{RNH}}_{2}↔{\mathrm{NH}}_{4}^{+}+R_{2}{\mathrm{NCOO}}^{-},$$

(3) $${\mathrm{CO}}_{2}+2R_{2}\mathrm{NH}↔R_{2}{\mathrm{NH}}_{2}^{+}+R_{2}{\mathrm{NCOO}}^{-},$$

(4) $${\mathrm{CO}}_{2}+2R_{3}N↔R_{4}N^{+}+R_{2}{\mathrm{NCOO}}^{-}.$$

In the presence of water, the carbamate ion formed by the reaction of both CO_2_ and amine will further react with water molecules to form bicarbonate as described in Equation (5):

(5) $$R_{2}{\mathrm{NCOO}}^{-}+H_{2}O↔R_{2}{\mathrm{NH}}_{2}^{+}+2{\mathrm{HCO}}_{3}^{-}.$$

Otherwise, amine groups can also directly react with CO_2_ and water molecules to form bicarbonate, as describes in Equations (6)–(8):

(6) $${\mathrm{CO}}_{2}+{\mathrm{RNH}}_{2}+H_{2}O↔{\mathrm{RNH}}_{3}^{+}+{\mathrm{HCO}}_{3}^{-},$$

(7) $${\mathrm{CO}}_{2}+R_{2}\mathrm{NH}+H_{2}O↔R_{2}{\mathrm{NH}}_{2}^{+}+{\mathrm{HCO}}_{3}^{-},$$

(8) $${\mathrm{CO}}_{2}+R_{3}N+H_{2}O↔R_{3}{\mathrm{NH}}^{+}+{\mathrm{HCO}}_{3}^{-}.$$

Generally speaking, water vapor can act as a free base, resulting in the formation of bicarbonate. In the presence of water one amine group could theoretically react with one CO_2_ molecule whereas two amine molecules are required to bind one molecule of CO_2_ under dry conditions. Therefore, in the presence of water vapor, the sensing performance of CO_2_ capacitive sensors based on PEI@silica adsorbents increases significantly due to the fact that one mole of CO_2_ can be adsorbed on one mole of amine. 

It is worth mentioning that the CO_2_ adsorption process on amine functionalized silica nanoparticles follows an adsorption–diffusion mechanism [50]. The adsorption of CO_2_ on the PEI surface is dominated by thermodynamics. However, the CO_2_ diffusion process is controlled by kinetics, which is an important factor in the adsorption process. Both mechanisms determine the CO_2_ adsorption on an amine functionalized silica. At lower temperature, in our case T < 46 °C, the adsorption of CO_2_ is mostly thermodynamically dominated by the surface layer. The adsorption capacity reflected by the capacitance change is low due to the lower molecular kinetic energy and the higher amine-based polymer viscosity. It is believed that PEI agglomerates in the sensing layer bulk, in a liquid-like phase, around and in between silica nanoparticles, generates strong diffusion limitations to CO_2_ into the sensing layer by encountering a high resistance. Therefore, only the amine adsorption sites on the upper part of the sensing layer are available for adsorbing CO_2_. As the operating temperature of CO_2_ sensor is increased from 46 to 60 °C, the molecular kinetic energy is gradually increased on the one side. On the other side, the viscosity of the amine-based polymer is decreased, and the PEI chains expand within the sensing layer. In general, as temperature increases, diffusion through the PEI phase improves, which facilitates the uptake of CO_2_ [51]. Hence, the CO_2_ molecules can diffuse easily and with less resistance into the bulk of the sensing layer. Therefore, more amine adsorption sites of PEI are available and accessible to adsorb CO_2_ molecules. Additionally, in the presence of enough water vapor molecules, the CO_2_ sensing performance is enhanced due to the fact that one mole of CO_2_ can be adsorbed on one mole of amine. Based on our experimental observation, if the operating temperature of the CO_2_ sensor is higher than 70 °C, the capacitance change becomes smaller. Thermodynamics may play a more important role, thus resulting in a decrease of the sorption capacity with increasing temperature. This observation can be explained either by the adsorption and desorption mechanism taking place simultaneously or by the evaporation of amine groups from the sensing layer.

#### 3.4.2. Long-Term Stability of a CO_2_ Gas Sensor

**Figure 8:**
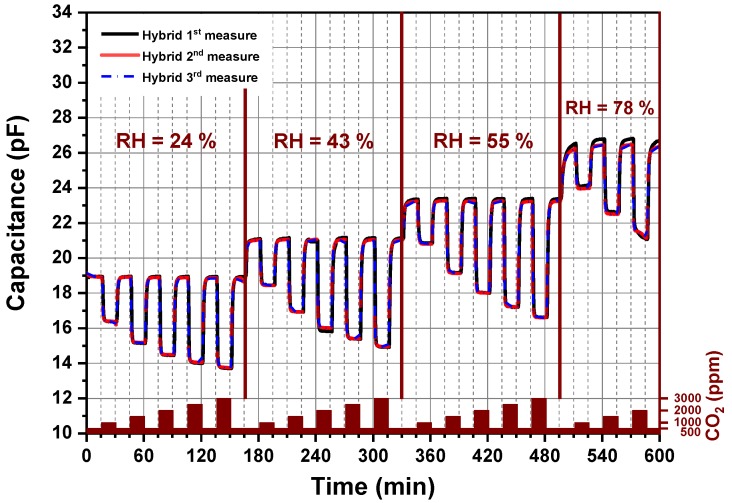
Long-term stability of the CO_2_ sensor: Each measurement, 1st, 2nd and 3rd, was performed by heating the sensor to 62 °C over a period of three weeks.

A long-term stability of amine functionalized silica nanoparticles under different CO_2_ concentrations and relative humidity levels permits a further estimation of the stability of the developed chemical CO_2_ sensor. As seen in Figure 8, the CO_2_ sensor device was investigated by cyclic exposure of the sensor to different CO_2_ concentrations in the presence of different humidity levels. Each cycle lasts 10 h. Each measurement, the 1st, 2nd and 3rd, was realized after 168 h at 62 °C over a period of three weeks. The capacitive CO_2_ sensor presents reproduceable results as the 1st, 2nd and 3rd cycles yielded identical measurements. Characterization of the hybrid organic–inorganic material over multiple adsorption–desorption cycles using mixed synthetic and CO_2_ gases under different humidity conditions showed that hybrid material present an enhanced stability in comparison to the published work related to sensing CO_2_ at temperatures lower than 60 °C because almost no one of them reported a clear statement about the sensor stability [14,17,20,31]. Furthermore, these materials offer the highest reported sensing capacities to date for a CO_2_ indoor application, giving higher capacities as compared to polysiloxane-based sensing material [34]. 

The realized work demonstrates and proves that silica-supported amine materials are promising adsorbents for a direct sensing of CO_2_ from our surrounding environment such as ambient air. 

## 4. Conclusions

In summary, we have introduced the impregnation method to functionalize amine on silica nanoparticles. The use of PEI@silica as a sensing material afforded to fabricate a new and high-performance chemical sensor for CO_2_. The sensor performance is optimal at low operating temperatures, that is, between 38 °C and 65 °C and at atmospheric pressure. The designed chemical sensor possesses excellent reversible sensing properties toward CO_2_, rapid response and recovery times, low power and low fabrication cost. We believe that PEI-modified silica material is a promising way to selectively and reversibly detect CO_2_. The CO_2_ sensor signal, depicted in the operating temperature between 38 °C and 65 °C, indicates that the diffusion of CO_2_ through the PEI phase improves by increasing the temperature. Hence, the number of accessible amine sites in the hybrid materials to CO_2_ molecules increase, thus increasing the CO_2_ adsorption capacity. Overall, the present study has demonstrated the first successful combination of an organic polymeric amine, PEI, and inorganic nanoparticles silica for sensing CO_2_. We anticipate that the employed method to functionalize the inorganic nanoparticles with amine groups is efficient and suited for wide application related to CO_2_ monitoring. We have further demonstrated the first chemical CO_2_ sensor with high long-term stability. Further developments of efficient sensing material for monitoring CO_2_ is necessary, especially to understand the effect of humidity to limit the sensor cross-sensitivity.

## Figures and Tables

**Table 1:** Measured properties of silica nanoparticles before and after loading with PEI.

Materials	S_BET_ (M^2^ G^−1^)	V_p_ (CM^3^ G−^1^)
PURE SILICA	189.74	0.741
PEI@SILICA (*100 NM*)	24.1	0.0058
